# Generation of Human Induced Pluripotent Stem Cell (hiPSC)-Derived Astrocytes for Amyotrophic Lateral Sclerosis and Other Neurodegenerative Disease Studies

**DOI:** 10.21769/BioProtoc.4936

**Published:** 2024-02-20

**Authors:** Katarina Stoklund Dittlau, Abinaya Chandrasekaran, Kristine Freude, Ludo Van Den Bosch

**Affiliations:** 1 Department of Neurosciences, Experimental Neurology, and Leuven Brain Institute, KU Leuven – University of Leuven, Leuven, Belgium; 2 Laboratory of Neurobiology, VIB Center for Brain & Disease Research, Leuven, Belgium; 3 Department of Veterinary and Animal Sciences, Faculty of Health and Medical Sciences, University of Copenhagen, Frederiksberg, Denmark

**Keywords:** Astrocyte, Human induced pluripotent stem cell, Neurodegeneration, Amyotrophic lateral sclerosis, Small-molecule differentiation

## Abstract

Astrocytes are increasingly recognized for their important role in neurodegenerative diseases like amyotrophic lateral sclerosis (ALS). In ALS, astrocytes shift from their primary function of providing neuronal homeostatic support towards a reactive and toxic role, which overall contributes to neuronal toxicity and cell death. Currently, our knowledge on these processes is incomplete, and time-efficient and reproducible model systems in a human context are therefore required to understand and therapeutically modulate the toxic astrocytic response for future treatment options. Here, we present an efficient and straightforward protocol to generate human induced pluripotent stem cell (hiPSC)-derived astrocytes implementing a differentiation scheme based on small molecules. Through an initial 25 days, hiPSCs are differentiated into astrocytes, which are matured for 4+ weeks. The hiPSC-derived astrocytes can be cryopreserved at every passage during differentiation and maturation. This provides convenient pauses in the protocol as well as cell banking opportunities, thereby limiting the need to continuously start from hiPSCs. The protocol has already proven valuable in ALS research but can be adapted to any desired research field where astrocytes are of interest.

Key features

• This protocol requires preexisting experience in hiPSC culturing for a successful outcome.

• The protocol relies on a small molecule differentiation scheme and an easy-to-follow methodology, which can be paused at several time points.

• The protocol generates >50 × 10^6^ astrocytes per differentiation, which can be cryopreserved at every passage, ensuring a large-scale experimental output.

**Graphical overview**


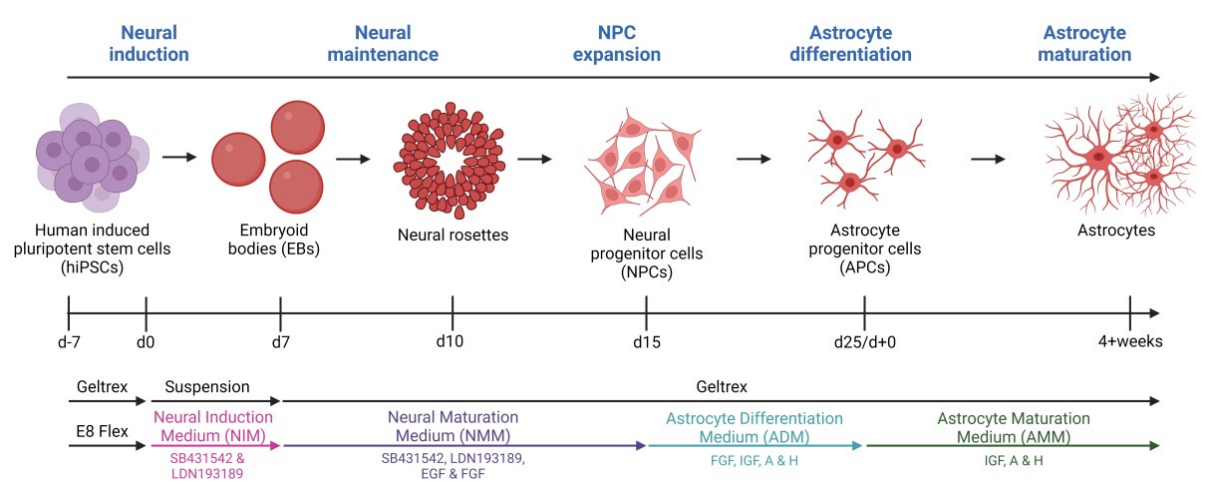


## Background

Neurodegenerative diseases affect millions of people worldwide and as the average population age increases, there is a corresponding rise in the number of patients. Amyotrophic lateral sclerosis (ALS) is one of these neurodegenerative diseases. ALS, the most prevalent motor neuron disorder among adults, affects approximately 2 out of 100,000 individuals across a wide age range, encompassing cases from teenagers to the elderly [1]. Ten percent of cases are caused by inherited familial mutations, while 90% have no family history and are therefore classified as sporadic [2]. Hallmarks of ALS include toxic protein aggregations, axonal transport impairments, DNA damage, and glial reactivity, leading to extensive motor neuron death [3–7]. This causes muscle atrophy, paralysis, and death of patients typically within 2–5 years after symptom onset, and currently, there is no cure [1]. As with many other neurodegenerative diseases, the focus has been on unraveling the underlying disease mechanisms behind the apparent (motor)neuronal cell death; however, the widespread glial reactivity has recently resulted in a shift from the neurocentric perspective towards increased appreciation of the role of glial cells. Astrocytes are shown to be key players in neurodegeneration [8]. As one of the most abundant glial cell types in the central nervous system, astrocytes govern the support and homeostatic maintenance of neurons and their surroundings [9]. Under physiological conditions, astrocytes have many functions including neurotransmitter modulation, nutritional distribution, ion, pH, and water homeostasis, blood–brain barrier regulation, and trophic support [9,10]. However, in ALS and other neurodegenerative diseases, astrocytes lose these supportive characteristics and take on a more toxic reactive role [10]. Considerable knowledge about the pathophysiology of ALS involving astrocytes has been gained through the use of animal models. However, it is important to acknowledge that, like all models, they come with their inherent limitations [10]. Animal models often rely on overexpression of human mutant genes, which despite showing various disease-relevant mechanisms, often fail to translate to a human context [11]. Importantly, overexpression models also exclude the large and important group of sporadic patients. Furthermore, human astrocytes exhibit larger sizes, more intricate branching structures, and a greater extent of synapse interactions compared to their rodent counterparts [12–14]. As a result, the field of animal research necessitates reinforcement from human in vitro models, and human-induced pluripotent stem cells (hiPSCs) present as highly promising candidates. With their ability for self-renewal and indefinite proliferation, as well as their possibility to generate any cell type, they hold significant potential. Several protocols for generating hiPSC-derived astrocytes exist, but many of the protocols are complex and require long timelines to reach the state of full astrocyte differentiation [15–21]. Our protocol is based on a 25-day-long differentiation followed by a 4+ week maturation. The differentiation is based on a dual inhibition of SMAD signaling pathway with the introduction of 3D culturing [22] to generate neural progenitor cells (NPCs) and a modified astrocyte differentiation protocol from Shaltouki et al. [23] to generate astrocytes [24,25]. After four weeks of maturation, > 95% of the hiPSC-derived astrocyte population is positive for typical astrocyte markers (S100β, AQP4, SOX9, and ALDH1L1) [24]. Importantly, the hiPSC-derived astrocytes retain their morphology, marker expression, and functionality, when cocultured with hiPSC-derived motor neurons [24].

## Materials and reagents

**Biological materials**

1. Human induced pluripotent stem cells (hiPSCs) (generated in-house [5])

**Reagents**

1. Essential 8^TM^ Flex medium kit (E8 Flex medium) (Thermo Fisher Scientific, Gibco, catalog number: A28583-01)
2. Geltrex^TM^ Matrix (Geltrex) (Thermo Fisher Scientific, Gibco, catalog number: A1413301)
3. DMEM/F-12 +Lglut, +HEPES (DMEM/F-12) (Thermo Fisher Scientific, Gibco, catalog number: 11330032)
4. Penicillin-Streptomycin (Pen/Strep) (5,000 U/mL) (Thermo Fisher Scientific, Gibco, catalog number: 15070063)
5. RevitaCell^TM^ supplement (100×) (Thermo Fisher Scientific, Gibco, catalog number: A2644501)
6. DPBS, no calcium, no magnesium (Thermo Fisher Scientific, Gibco, catalog number: 14190144)
7. Collagenase type IV powder (Thermo Fisher Scientific, Gibco, catalog number: 17104019)
8. SB431542 (Tocris Bioscience, catalog number: 1614; product format: 10 mM in ethanol)
9. LDN193189 (Stemgent, catalog number: 04-0074-02; product format: 10 mM in solution)
10. Neurobasal^TM^ medium (Thermo Fisher Scientific, Gibco, catalog number: 21103049)
11. B-27^TM^ supplement minus vitamin A (50×) (Thermo Fisher Scientific, Gibco, catalog number: 12587-010)
12. N-2 Supplement (100×) (Thermo Fisher Scientific, Gibco, catalog number: 17502048)
13. L-Glutamine (200 mM) (Thermo Fisher Scientific, Gibco, catalog number: 25030024)
14. Aqua ad iniectabilia (injectable water) (B. Braun, catalog number: 2351744)
15. Recombinant murine FGF-basic (FGF) (Peprotech, catalog number: 450-33; product format: 10 µg/mL in DPBS)
16. Recombinant human epidermal growth factor (EGF) (ProSpec, catalog number: CYT-217; product format: 100 µg/mL in injectable water)
17. Accutase^®^ solution (Sigma-Aldrich, catalog number: A6964)
18. Fetal bovine serum (FBS) (Thermo Fisher Scientific, Gibco, catalog number: 10270106)
19. Dimethyl sulfoxide (DMSO) (Sigma-Aldrich, catalog number: D2650)
20. MEM non-essential amino acids solution (NEAA) (100×) (Thermo Fisher Scientific, Gibco, catalog number: 11140050)
21. L-Ascorbic acid (Sigma-Aldrich, catalog number: A4403; product format: 200 µM in injectable water)
22. Recombinant human IGF-1 (IGF) (Peprotech, catalog number: 100-11; product format: 100 µg/mL in injectable water)
23. Human Activin A recombinant protein (A) (Thermo Fisher Scientific, Gibco, catalog number: PHC9564; product format: 10 µg/mL in DPBS)
24. Recombinant human Heregulin β-1 (H) (Peprotech, catalog number: 100-03, product format: 250 µg/mL in injectable water)
25. Sodium pyruvate (100 mM) (Thermo Fisher Scientific, Gibco, catalog number: 11360070)
26. Trypan blue solution, 0.4% (Thermo Fisher Scientific, Gibco, catalog number: 15250061)
27. Ethanol absolute ≥ 99.8% (VWR, catalog number: 20821.296)
28. Isopropanol, 99.5% (Thermo Fisher Scientific, catalog number: 184130010)

**Solutions**

1. E8 Flex medium (see Recipes)
2. Geltrex coating (see Recipes)
3. Collagenase type IV (10× and 1× solutions) (see Recipes)
4. Neural induction medium (NIM) (see Recipes)
5. Neural maturation medium (NMM) (see Recipes)
6. Astrocyte differentiation medium (ADM) (see Recipes)
7. Astrocyte maturation medium (AMM) (see Recipes)

**Recipes**

1. **E8 Flex medium**
  Thaw the frozen Essential 8^TM^ Flex supplement from the Essential 8^TM^ Flex medium kit at room temperature for approximately 1 h or at 2–8 °C overnight. Protect the supplement from light, as it is light sensitive. Mix the thawed supplement by gently inverting the vial a couple of times and then aseptically transfer the entire contents of the Essential 8^TM^ Flex supplement to the bottle of Essential 8^TM^ Flex basal medium. Swirl the bottle to mix. E8 Flex medium can be stored at 2–8 °C for up to two weeks.
  **Table:** 

Reagent	Final concentration	Volume
Essential 8^TM^ Flex basal medium	98%	500 mL
Essential 8^TM ^Flex supplement (50×)	2%	10 mL
Total	100%	510 mL
2. **Geltrex coating**
  It is important to keep all components at ≤ 2–8 °C during the preparation of the Geltrex coating to prevent premature solidification. To ensure this, first prepare 24.75 mL of 2–8 °C DMEM/F-12 in a 50 mL conical tube and then collect the Geltrex aliquot from -20 °C. Transfer approximately 0.5 mL of 2–8 °C DMEM/F-12 from the prepared 50 mL conical tube to the Geltrex aliquot, pipette up and down to dissolve the frozen aliquot, and transfer approximately 0.75 mL of the solution back to the 50 mL conical tube. Repeat the process a few times to transfer the entire content of the Geltrex aliquot to the 50 mL conical tube. Mix well. Geltrex coating can be stored at 2–8 °C for one week.
  **Table:** 

Reagent	Final concentration	Volume
DMEM/F-12	99%	24.75 mL
Geltrex	1%	250 µL
Total	100%	25 mL
3. **Collagenase type IV (10× and 1× solutions)**
  First, prepare a stock concentration (10×): Dilute 1 g of collagenase type IV powder in 100 mL of DMEM/F-12 and filter sterilize. Aliquot the 10× solution in 10 mL/aliquot. Next, prepare the working concentration (1×): Dilute 10 mL of the stock concentration (10×) with 90 mL of DMEM/F-12 to make a 1× solution and filter sterilize. Aliquot the 1× solution in 12.5 mL/aliquot. Both 10× and 1× aliquots can be stored at -20 °C for ≤ 6 months. Bring collagenase type IV (1×) to room-temperature before use.
  **Table:** 

Reagent	Final concentration	Quantity or Volume
DMEM/F-12	100%	100 mL
Collagenase type IV (powder)	10 mg/mL (10×)	1 g
DMEM/F-12	90%	90 mL
Collagenase type IV (10×)	1 mg/mL (1×)	10 mL
4. **Neural induction medium (NIM): d0-d6**
  Prepare ~500 mL of bulk solution of E8 Flex medium and Pen/Strep and filter sterilize. E8 Flex + Pen/strep can be stored at 2–8 °C for two weeks. To prepare NIM, make an aliquot of the required volume for the day of E8 Flex + Pen/Strep solution and add SB431542 and LDN193189 fresh on the day of use. Filter sterilize and bring the NIM solution to room temperature before use.
  **Table:** 

Reagent	Final concentration	Volume
E8 Flex medium	100%	500 mL
Pen/Strep	1%	5 mL
SB431542	10 µM	see note
LDN193189	0.1 µM	see note
5. **Neural maturation medium (NMM): d7-d15**
  Prepare > 200 mL of bulk solution of basic medium (DMEM/F12, neurobasal medium, Pen/Strep, B-27 minus vitamin A, N-2, and L-Glutamine) and filter sterilize. Basic medium can be stored at 2–8 °C for four weeks. To prepare NMM, make an aliquot of the required volume for the day of basic medium solution and add SB431542, LDN193189, FGF, and EGF fresh on the day of use. Filter sterilize and bring the NMM solution to 37 °C before use.
  **Table:** 

Reagent	Final concentration	Volume (for 200 mL)
DMEM/F-12	47.5%	95 mL
Neurobasal medium	47.5%	95 mL
Pen/Strep	1%	2 mL
B-27 minus vitamin A	2%	4 mL
N-2	1%	2 mL
L-Glutamine	1%	2 mL
SB431542	10 µM	see note
LDN193189	0.1 µM	see note
FGF	10 ng/mL	see note
EGF	10 ng/mL	see note
6. **Astrocyte differentiation medium (ADM): d16-d25**
  Prepare >200 mL of bulk solution of basic medium (neurobasal medium, Pen/Strep, N-2, NEAA, and L-Ascorbic acid) and filter sterilize. Basic medium can be stored at 2–8 °C for four weeks. To prepare ADM, make an aliquot of the required volume for the day of basic medium solution and add FGF, IGF, A, and H fresh on the day of use. Filter sterilize and bring the ADM solution to 37 °C before use.
  **Table:** 

Reagent	Final concentration	Volume (for 200 mL)
Neurobasal medium	97%	194 mL
Pen/Strep	1%	2 mL
N-2	1%	2 mL
NEAA	1%	2 mL
L-Ascorbic acid (200 µM)	0.8 µM	800 µL
FGF	10 ng/mL	see note
IGF	200 ng/mL	see note
A	10 ng/mL	see note
H	10 ng/mL	see note
7. **Astrocyte maturation medium (AMM): d25+**
  Prepare 500 mL of bulk solution of basic medium (DMEM/F12, neurobasal medium, Pen/Strep, N-2, NEAA, L-Ascorbic acid, L-Glutamine, sodium pyruvate, and FBS) and filter sterilize. Basic medium can be stored at 2–8 °C for four weeks. To prepare AMM, make an aliquot of the required volume for the day of basic medium solution and add IGF, A, and H fresh on the day of use. Filter sterilize and bring the AMM solution to 37 °C before use.
  **Table:** 

Reagent	Final concentration	Volume (for 500 mL)
DMEM/F-12	46.3%	231.5 mL
Neurobasal medium	46.3%	231.5 mL
Pen/Strep	1%	5 mL
N-2	1%	5 mL
NEAA	1%	5 mL
L-Ascorbic acid (200 µM)	0.8 µM	2 mL
L-Glutamine	1%	5 mL
Sodium pyruvate	1%	5 mL
FBS	2%	10 mL
IGF	200 ng/mL	see note
A	10 ng/mL	see note
H	10 ng/mL	see note
**Laboratory supplies**
  1. Cell culture flasks, 25 cm^2^, cell-repellent surface (T25 non-adherent flask) (Greiner Bio-One, catalog number: 690980)
  2. Cell culture multi-well plates (6-well plate) (Greiner Bio-One, catalog number: 657160)
  3. 25 mL sterile reservoirs (Thermo Fisher Scientific, catalog number: 95128095) or 50 mL sterile reservoirs (InvitroLab, catalog number: IV-6002)
  4. Cell scrapers (TH Geyer, catalog number: 7696760)
  5. 15 mL conical tubes (TH Geyer, catalog number: 7696714)
  6. 50 mL conical tubes (Greiner Bio-One, catalog number: 227261)
  7. 150 mL vacuum filtration devices, pore 0.22 µm (Jet Biofil, catalog number: FCF010004)
  8. 500 mL vacuum filtration devices, pore 0.22 µm (Jet Biofil, catalog number: FPE204500)
  9. 2 mL cryovials (Maxxline, catalog number: MLC2B)
  10. 5 mL serological pipettes (Greiner Bio-One, catalog number: 606180)
  11. 10 mL serological pipettes (Greiner Bio-One, catalog number: 607180)
  12. 25 mL serological pipettes (Greiner Bio-One, catalog number: 760160-TRI)
  13. Sterile PES syringe filters (Thermo Fisher Scientific, catalog number: 15206869)
  14. 50 mL 3-part syringes (Chirana T. Injecta, catalog number: CH03050LL)
  15. 10 µL pipette tips (TH Geyer, catalog number: 7695881)
  16. 20 µL pipette tips (TH Geyer, catalog number: 7695882)
  17. 200 µL pipette tips (TH Geyer, catalog number: 7695884)
  18. 1,250 µL pipette tips (TH Geyer, catalog number: 7695887)
  19. Countess^TM^ cell counting chamber slides (Thermo Fisher Scientific, Invitrogen, catalog number: C10283)

## Equipment

1. NordicSafe^®^ Class II biological safety cabinet (ESCO, catalog number: NC2-L)
2. CellXpert^®^ C170i CO_2_ incubator (Eppendorf, catalog number: 6734)
3. EVOS^TM^ XL Core inverted microscope (objectives: 4×, 10×, 20×) (Thermo Fisher Scientific, catalog number: AMEX1000)
4. Laboratory centrifuge with rotors for 15 and 50 mL conical tubes (Biosan, catalog number: LMC-3000)
5. Water bath (37 °C) (Julabo, catalog number: TW8)
6. Finnpipette^TM^ F2 GLP pipetting kit 2 (Thermo Fisher Scientific, catalog number: 11835850)
7. Pipetboy acu 2 (Integra, catalog number: 1550179)
8. Mr. Frosty^TM^ freezing container (Thermo Fisher Scientific, catalog number: 5100-0001)
9. Countess^TM^ II FL automated cell counter (Thermo Fisher Scientific, catalog number: AMQAF1000)
10. Racks
11. Liquid nitrogen (N_2_) tank
12. Freezer (-20 °C)
13. Refrigerator (2–8 °C)

## Procedure

- **Neural induction**
  1. Plate hiPSCs on Geltrex-coated 6-well plates (Table 1) in 2 mL/well of E8 Flex medium and expand according to standard protocol. See Recipes 1 and 2.
*Note: The use of Geltrex can be replaced by Matrigel during the entire protocol.*
  2. After a minimum of seven days as iPSCs, cell lines are prepared for neural induction when reaching 70%–90% confluence (day 0).
*Notes:*
    - *Use a full 6-well plate per cell line to start one differentiation.*
    - *To avoid excessive weekend work, start day 0 (d0) on a Monday.*
  3. Remove spent E8 Flex medium, wash cells once with 1 mL/well of DPBS, and incubate with 1 mL/well of room-temperature collagenase type IV (1×) for 10–20 min at 37 °C with 5% CO_2_ to dissociate the colonies. See Recipe 3.
*Note: When ready, iPSC colonies will lift and curl up around the borders. Larger colonies might require longer incubation time. After 10 min incubation, check under light microscope every 5 min. Maximum collagenase type IV (1×) incubation time: 60 min.*
  4. After incubation, remove the spent collagenase and add 1 mL/well of room-temperature E8 Flex medium, gently scrape the loosened colonies with a cell scraper, and use a P1000 pipette to transfer the cell suspension to individual 15 mL conical tubes.
*Notes:*
    - *Use one 15 mL conical tube per 6-well plate.*
    - *Avoid excess pipetting to sustain clumps of colonies.*
    - *If needed, use 1 mL/well of fresh E8 Flex medium to gently flush around the borders of the well to collect remaining cells.*
  5. Incubate the conical tube at 37 °C with 5% CO_2_ for 15 min to allow the clusters to sediment.
  6. After incubation, remove supernatant.
  7. Carefully dissolve cell pellet in 10 mL of room-temperature NIM (see Recipe 4) by pipetting up and down a few times and transfer the cell suspension to a T25 non-adherent flask.
**Critical:** Do not pipette up and down too much to sustain cell clumps.
*Note: Mark as passage 0 (P0).*
  8. Check the cell density under a light microscope and incubate the flask at 37 °C with 5% CO_2_.
  9. Perform a medium change with 10 mL/flask of room-temperature NIM on day 1 (d1), d2, and d4.
    When changing medium, place the T25 non-adherent flask in an upright position in a 25 or 50 mL sterile reservoir in the incubator and allow cells to sediment for 5–10 min (Figure 1A). Carefully transfer the flask in its upright position into the biological safety cabinet and remove approximately 9 mL of spent medium to allow the cells to remain covered in a small volume of medium. Add 10 mL of fresh room-temperature NIM per flask.
*Notes:*
    - *Three to four days after the start of induction, embryoid bodies (EBs) are clearly visible (Figure 1B). EBs appear irregular around their borders for the first few days but take on a rounder form during the induction phase.*
    - *A cell shaker is not required during neural induction. If the EBs begin to adhere to each other, perform a gentle pipetting during medium changes.*
    See **Troubleshooting** if EBs attach to the bottom of the flask.
  **Figure 1.**
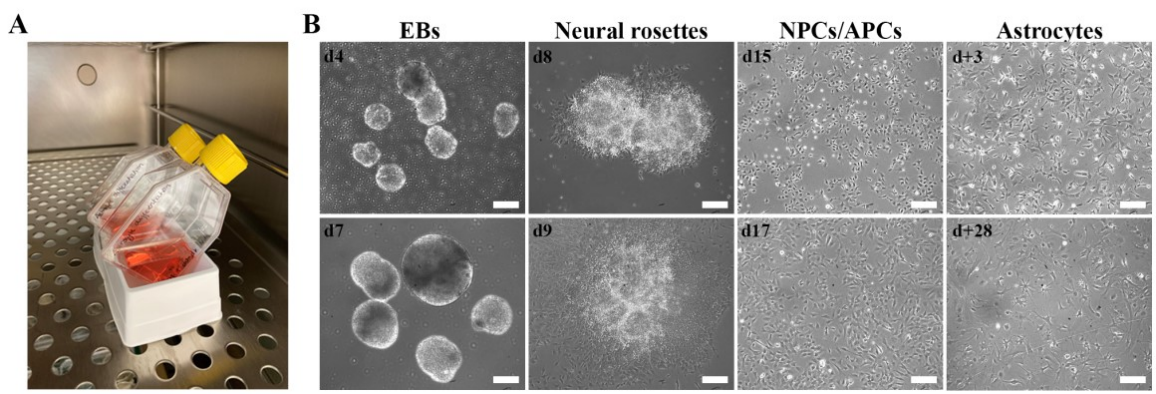
Cell morphology during the astrocyte differentiation protocol. A. Image illustrating the use of a sterile reservoir for flask support to allow embryoid body (EB) sedimentation during medium changes. B. Brightfield images of EBs, cell morphologies, and optimal confluence during the astrocyte differentiation protocol. Scale bar: 200 µm. d+ refers to days of astrocyte maturation. The use of patient fibroblasts for the generation of hiPSCs was approved by the ethics committee of University Hospital Leuven (number S50354 and S63792).
  **Table 1.** Differentiation step and assay overview

Differentiation step/assay	Plate format	Seeding density	Optimal seeding time point	Medium change volume	Geltrex-coating volume	Dissociation/fixation reagent volume (e.g., accutase)
Start of differentiation	6-well plate	70%–90% confluent hiPSCs	Day 0 (d0)	2 mL/well	1 mL/well	1 mL/well
Neural induction	T25 non-adherent flask	NA	Day 0 (d0)	10 mL/flask	NA	NA
Neural maintenance	6-well plate	NA	Day 7 (d7)	2 mL/well	1 mL/well	1 mL/well
Neural expansion/thawing	6-well plate	70%–100% confluence	See Procedure C and D	2 mL/well	1 mL/well	1 mL/well
Astrocyte differentiation	6-well plate	90%–100% confluence	Day 16 (d16)	2 mL/well	NA	NA
Astrocyte maturation/expansion/thawing	6-well plate	70%–100% confluence	See Procedure F–H	2 mL/well	1 mL/well	1 mL/well
Immunocytochemistry	24-well plate	30–50,000 cells/cm^2^	Two days before fixation	0.5 mL/well	0.5 mL/well	0.5 mL/well
Transmission electron microscopy	24-well plate	50,000 cells/cm^2^	>2 weeks before experiment	0.5 mL/well	0.5 mL/well	0.5 mL/well
Protein extraction	6-well plate	50,000 cells/cm^2^	>2 weeks before experiment	2 mL/well	1 mL/well	1 mL/well
RNA extraction	6-well plate	50,000 cells/cm^2^	>2 weeks before experiment	2 mL/well	1 mL/well	1 mL/well
Metabolic assays	12-well plate	32,000 cells/cm^2^	2–7 days before experiment	1 mL/well	0.75 mL/well	0.75 mL/well
- **Neural maintenance**
  1. On d7, plate EBs for neural rosette formation: prepare one full Geltrex-coated 6-well plate (Table 1) per T25 non-adherent flask and incubate at 37 °C with 5% CO_2_ for at least 30 min.
*Note: Geltrex-coated plates can be prepared the day before and incubated at 37 °C with 5% CO_2_ overnight.*
  2. After 30 min, remove spent Geltrex and add 1 mL/well of NMM (see Recipe 5). Incubate the plates at 37 °C with 5% CO_2_ to be ready for use.
  3. Transfer EBs in spent media to individual 15 mL conical tubes (one conical tube per T25 non-adherent flask) and incubate upright at 37 °C with 5% CO_2_ for 5–10 min to allow sedimentation of EBs.
  4. After the incubation, aspirate spent medium and add 1 mL/well of 37 °C NMM to the conical tube without pipetting up and down.
  5. Transfer 1 mL/well of EB suspension to the prepared Geltrex-coated 6-well plates with 1 mL/well of NMM so that each well contains 2 mL of NMM. Incubate the plate at 37 °C with 5% CO_2_.
*Note: Make sure to evenly distribute the EBs among the different wells and finish by carefully rocking the plate to facilitate even EB dispersal.*
  6. Change medium daily with 37 °C NMM until d10.
*Note: At d8–d9, small colonies, each with 1–10 neural rosettes containing NPCs, will be visible (Figure 1B).*
  7. On d10, neural rosettes are clearly visible and ready for passaging (P1) and NPC expansion.
  8. Prepare one Geltrex-coated 6-well plate per 6-well plate with cells and incubate at 37 °C with 5% CO_2_ for at least 30 min.
*Note: Geltrex-coated plates can be prepared the day before and incubated at 37 °C with 5% CO_2_ overnight.*
  9. After 30 min, remove spent Geltrex and add 1 mL/well of NMM + 5 µL/mL RevitaCell^TM^ supplement. Incubate the plates at 37 °C with 5% CO_2_ to be ready for use.
  10. Incubate neural rosettes with 1 mL/well of room-temperature accutase for 4 min at 37 °C with 5% CO_2 _to detach the neural rosettes containing NPCs.
  11. After the incubation, add 1 mL/well of 37 °C NMM to inactivate the accutase.
  12. Gently scrape the cells with a cell scraper and transfer cell suspension to a 15 mL conical tube.
*Note: As it is not recommended to centrifuge with volumes below 3 mL for 15 mL conical tubes, increase the volume above 3 mL by adding more NMM if needed.*
  13. Centrifuge at 145× *g* for 4 min at room temperature.
  14. Remove supernatant, add 1 mL/well of 37 °C NMM + 5 µL/mL RevitaCell^TM^ supplement, and carefully pipette up and down to dissolve the cell pellet.
  15. Transfer 1 mL/well of cell suspension to the prepared Geltrex-coated 6-well plates with 1 mL/well of NMM + 5 µL/mL RevitaCell^TM^ supplement so that each well contains 2 mL of NMM + 5 µL/mL RevitaCell^TM^ supplement.
*Note: To evenly distribute the NPCs, carefully rock the plate from side to side.*
  16. Examine cell distribution under the light microscope and incubate the plate at 37 °C with 5% CO_2_.
  17. Perform medium change with 2–6 mL/well of 37 °C NMM on d11, followed by every second day until day 16.
*Note: NPCs are usually 100% confluent on d11 (Friday), but do not require passaging. Weekend work can be avoided by giving 4–6 mL/well of 37 °C NMM on d11 (Friday), followed by passaging (P2) and cryopreservation on d14 (Monday). Standard medium changes are 2 mL/well every second day.*
**Pause point:** On d12–d14, NPCs are passaged (P2) and/or cryopreserved one time. If desired, the protocol can be paused and restarted at this time point in the differentiation. Always change the medium the day after passaging/thawing NPCs.
**Critical:** Make sure the NPCs are 90%–100% confluent when changing to ADM (see Recipe 6) on d16 (Figure 1B). ADM is harsh on the cells and causes extensive cell death. The NPCs require cell–cell contact to survive day 16–25 (d16–d25/d+0) of the differentiation protocol.
- **NPC expansion**
  1. On d12–d14, passage NPCs 1:3–1:6 once to enhance cell expansion (P2).
*Note: Passage ratio depends on cell confluence and growth rate, as it is important to have 90%–100% confluent cells by d16. Of a full 6-well plate, authors recommend passaging one well 1:3–1:6 and cryopreserving the remaining five wells (one cryovial/well).*
  2. Prepare Geltrex-coated 6-well plates (Table 1) and incubate at 37 °C with 5% CO_2_ for at least 30 min.
*Note: Geltrex-coated plates can be prepared the day before and incubated at 37 °C with 5% CO_2_ overnight.*
  3. After 30 min, remove spent Geltrex and add 1 mL/well of NMM + 5 µL/mL RevitaCell^TM^ supplement. Incubate the plates at 37 °C with 5% CO_2_ to be ready for use.
  4. Remove spent NMM, wash cells once with DPBS, and incubate with 1 mL/well of room-temperature accutase for 4 min at 37 °C with 5% CO_2_.
  5. After the incubation, gently tap the side of the plate to check if cells readily detach.
*Note: If cells do not detach easily, incubate for one more minute and repeat step C5.*
  6. Add 1 mL/well of 37 °C NMM to inactivate the accutase.
  7. Gently flush along the sides of the well to detach remaining NPCs and transfer cell suspension to a 15 mL conical tube.
*Notes:*
    - *If not all cells detach after 5 min incubation with accutase, use a cell scraper to collect remaining cells.*
    - *As it is not recommended to centrifuge with volumes below 3 mL for 15 mL conical tubes, increase the volume above 3 mL by adding more NMM if needed.*
  8. Centrifuge at 145× *g* for 4 min at room temperature.
  9. Remove supernatant and continue with step C10 for passaging or step C11 for cryopreservation.
  10. For passaging:
    - Add 1 mL/well of 37 °C NMM + 5 µL/mL RevitaCell^TM^ supplement and carefully pipette up and down to dissolve the cell pellet.
    - Transfer 1 mL/well of cell suspension to the prepared Geltrex-coated 6-well plates with 1 mL/well of NMM + 5 µL/mL RevitaCell^TM^ supplement, so that each well contains 2 mL of NMM + 5 µL/mL RevitaCell^TM^ supplement.
    - To evenly distribute the NPCs, carefully rock the plate from side to side.
    - Examine cell distribution under the light microscope and incubate the plate at 37 °C with 5% CO_2_.
    - The following day, perform a medium change with 2 mL/well of 37 °C NMM followed by every second day until d16.
  11. For cryopreservation:
    - Add 1 mL/vial of room-temperature freezing medium (90% FBS and 10% DMSO) and carefully pipette up and down to dissolve the cell pellet.
    - Transfer 1 mL of cell suspension to prelabeled cryovials, transfer vials to a Mr. Frosty with isopropanol, and incubate at -80 °C overnight.
    - The following day, transfer vials to liquid N_2_ storage for long-term cryopreservation.
- **Thawing NPCs**
  1. Cryopreserved NPCs (d12–d14) should be thawed and continued on the same day of the differentiation protocol.
*Note: For example, cryopreserved d14 NPCs are thawed and continued on day 14 of the protocol.*
  2. Prepare Geltrex-coated 6-well plates (Table 1) (one well/cryovial) and incubate at 37 °C with 5% CO_2_ for at least 30 min.
*Note: Geltrex-coated plates can be prepared the day before and incubated at 37 °C with 5% CO_2_ overnight.*
  3. Per cryovial, prepare 15 mL conical tubes with 9 mL of 37 °C basic NMM (without growth factors).
  4. After 30 min, remove spent Geltrex and add 1 mL/well of NMM (with growth factors) + 5 µL/mL RevitaCell^TM^ supplement. Incubate the plates at 37 °C with 5% CO_2_ to be ready for use.
  5. Remove the cryovial from liquid N_2_ storage and thaw for approximately 1 min in a 37 °C water bath until a small *pea-sized* ice clump is left.
  6. Transfer ~0.5 mL of 37 °C basic NMM (without growth factors) from the prepared 15 mL conical tube to the cryovial, pipette up and down to dissolve remaining ice, and transfer ~1 mL of cell suspension back to the 15 mL conical tube. Repeat the process a few times to transfer the entire content of the cryovial to the 15 mL conical tube.
  7. Centrifuge at 145× *g* for 4 min at room temperature.
  8. Remove supernatant and add 1 mL/well of 37 °C NMM (with growth factors) + 5 µL/mL RevitaCell^TM^ supplement and carefully pipette up and down to dissolve the cell pellet.
  9. Transfer 1 mL/well of cell suspension to the prepared Geltrex-coated plates with 1 mL/well of NMM (with growth factors) + 5 µL/mL RevitaCell^TM^ supplement, so that each well contains 2 mL of NMM (with growth factors) + 5 µL/mL RevitaCell^TM^ supplement. To evenly distribute the NPCs, carefully rock the plate from side to side.
  10. Examine cell distribution under the light microscope and incubate the plate at 37 °C with 5% CO_2_.
  11. Perform a medium change with 2 mL/well of 37 °C NMM (with growth factors) the next day, followed by every second day until d16.
- **Astrocyte differentiation**
  1. On d16, change medium to 2 mL/well of 37 °C ADM (see Recipe 6) to commence the conversion of NPCs to astrocyte progenitor cells (APCs) and induce astrocyte differentiation (Figure 1B).
  2. Change the ADM every second day until day 25 (d25/d+0), when a glial switch to astrocytes is expected to occur.
**Critical:** Do not passage cells during this period to ensure a sustained confluent layer of cells.
*Notes:*
    - *Medium changes can be avoided in the weekend if giving 4–6 mL/well of 37 °C ADM on a Friday.*
    - *Potential intracellular vacuolization and/or increased cell death are expected during this period.*
    See **Troubleshooting** if complete cell death occurs during d16–d25.
- **Astrocyte maturation**
  1. On d25, astrocyte maturation is commenced (d+0). Several options are available at this time point:
**Pause point:** On day 25 (d25/d+0), astrocytes can be cryopreserved. If desired, the protocol can be paused and restarted at this time point. To commence, follow Procedure G.
    - If large cell samples are desired (such as for protein and RNA extraction), passage and plate some of the cells in specified cell densities (Table 1) in AMM (see Recipe 7) to allow maturation of astrocytes for 4 weeks. Astrocytes are not passaged during this period and therefore become very confluent (~3D). Adjust AMM volume accordingly: 4–6 mL/well with every medium change (Monday, Wednesday, and Friday). Remaining d25/d+0 astrocytes can be plated for expansion and/or cryopreserved. To commence, follow Procedure G.
    - Create an astrocyte cell bank: Passage d25/d+0 astrocytes 1:6 for maturation and expansion. Every 4–7 days, passage astrocytes 1:6 over the time course of 14 days. After two weeks (d+14), cryopreserve astrocytes for future experiments (1–3 cryovials per confluent well of a 6-well plate). The protocol can be paused and restarted any day during the maturation. For future experiments, thaw d+14 astrocytes, expand for one week, and plate according to recommended cell densities (Table 1). Change medium every second day. To commence, follow Procedure G.
*Notes:*
*i. For immunocytochemistry experiments, plate cells two days before fixation to avoid excessive cell proliferation. Change medium the day after plating.*
*ii. Authors have observed astrocyte proliferation until week 6 of maturation.*
*iii. Astrocyte markers increase over the time course of 3–4 weeks of maturation. Approximately 95% of astrocytes at positive for astrocyte markers (SOX9, S100β, ALDH1L1, and AQP4) at week 4 of maturation (d+28). See Figure 2.*
  **Figure 2.**
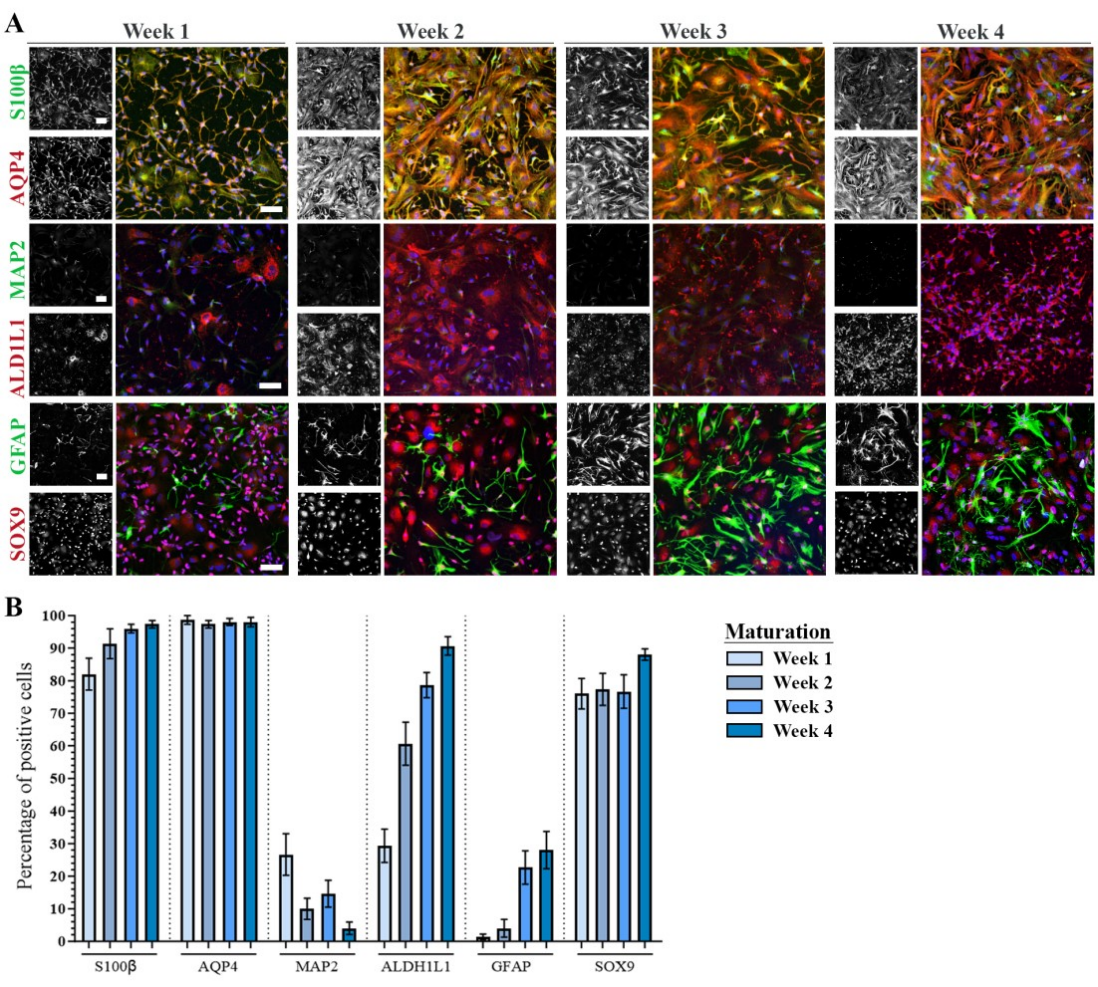
Astrocyte maturation verification with immunocytochemistry. A. Representative confocal images of human induced pluripotent stem cell (hiPSC)-derived astrocytes at week 1–4 of maturation stained with astrocyte-specific markers S100β, AQP4, ALDH1L1, GFAP, SOX9, and neuronal marker MAP2. Nuclei stained with DAPI (blue). Scale bar: 75 μm. B. Quantification of the number of antibody-positive cells during week 1–4 of maturation. Mean ± S.E.M. of three biological replicates (n = 15 images). The figure is modified from Stoklund Dittlau et al. [24] with permission under the Creative Commons Attribution 4.0 International License (http://creativecommons.org/licenses/by/4.0/).
- **Passaging and cryopreservation of astrocytes**
  1. Prepare Geltrex-coated plates (for recommended plate format see Table 1) and incubate at 37 °C with 5% CO_2_ for at least 30 min.
*Note: Geltrex-coated plates can be prepared the day before and incubated at 37 °C with 5% CO_2_ overnight.*
  2. After 30 min, remove spent Geltrex and add half of the volume of AMM + 5 µL/mL RevitaCell^TM^ supplement (for required volumes see Table 1). Incubate the plates at 37 °C with 5% CO_2_ to be ready for use.
  3. Remove spent ADM/AMM, wash cells once with DPBS, and incubate with room-temperature accutase (for required volumes see Table 1) for 4 min at 37 °C with 5% CO_2_.
  4. After the incubation, gently tap the side of the plate to check if cells readily detach.
*Note: If cells do not detach easily, incubate for one more min and repeat step G4.*
  5. Add 1:1 volume of 37 °C AMM to inactivate the accutase.
  6. Gently flush along the sides of the well to detach remaining astrocytes and transfer cell suspension to a 15 mL conical tube.
*Notes:*
    - *If not all cells detach after 5 min incubation with accutase, use a cell scraper to collect remaining cells.*
    - *As it is not recommended to centrifuge with volumes below 3 mL for 15 mL conical tubes, increase the volume above 3 mL by adding more AMM if needed.*
  7. Centrifuge at 145× *g* for 4 min at room temperature.
  8. Remove supernatant.
  9. For passaging:
    - Add the remaining half volume of 37 °C AMM + 5 µL/mL RevitaCell^TM^ supplement (for required volumes see Table 1) and carefully pipette up and down to dissolve the cell pellet.
    - Transfer cell suspension to the prepared Geltrex-coated plates with AMM + 5 µL/mL RevitaCell^TM^ supplement.
    - To evenly distribute the astrocytes, carefully rock the plate from side to side.
    - Examine cell distribution under the light microscope and incubate the plate at 37 °C with 5% CO_2_.
    - Perform medium change with 37 °C AMM the next day followed by every second day.
  10. For cryopreservation:
    - Add 1 mL/vial of room-temperature freezing medium (90% FBS and 10% DMSO) and carefully pipette up and down to dissolve the cell pellet.
    - Transfer 1 mL of cell suspension to prelabeled cryovials, transfer vials to a Mr. Frosty with isopropanol, and incubate at -80 °C overnight.
    - The following day, transfer vials to liquid N_2_ storage for long-term cryopreservation.
- **Thawing astrocytes**
  1. **Critical:** When thawing astrocytes, allow at least one week of recovering, passaging, and expansion in 6-well plates before plating for experiments.
*Note: Authors recommend to passage cells 1–2 times every 3–4 days before plating for experiments. Passaging enhances viability and culture purity.*
  2. Prepare Geltrex-coated 6-well plates (Table 1) (one well/cryovial) and incubate at 37 °C with 5% CO_2_ for at least 30 min.
*Note: Geltrex-coated plates can be prepared the day before and incubated at 37 °C with 5% CO_2_ overnight.*
  3. Per cryovial, prepare 15 mL conical tubes with 9 mL of 37 °C basic AMM (without growth factors).
  4. After 30 min, remove spent Geltrex and add 1 mL/well of AMM (with growth factors) + 5 µL/mL RevitaCell^TM^ supplement. Incubate the plates at 37 °C with 5% CO_2_ to be ready for use.
  5. Remove cryovial from liquid N_2_ storage and thaw for approximately 1 min in a 37 °C water bath until a small *pea-sized* ice clump is left.
  6. Transfer ~0.5 mL of 37 °C basic AMM (without growth factors) from the prepared 15 mL conical tube to the cryovial, pipette up and down to dissolve remaining ice, and transfer ~1 mL of cell suspension back to the 15 mL conical tube. Repeat the process a few times to transfer the entire content of the cryovial to the 15 mL conical tube.
  7. Centrifuge at 145× *g* for 4 min at room temperature.
  8. Remove supernatant and add 1 mL/well of 37 °C AMM (with growth factors) + 5 µL/mL RevitaCell^TM^ supplement and carefully pipette up and down to dissolve cell pellet.
  9. Transfer 1 mL/well of cell suspension to the prepared Geltrex-coated plates with 1 mL/well of AMM (with growth factors) + 5 µL/mL RevitaCell^TM^ supplement, so each well contains 2 mL of AMM (with growth factors) + 5 µL/mL RevitaCell^TM^ supplement.
*Note: To evenly distribute the astrocytes, carefully rock the plate from side to side.*
  10. Examine cell distribution under the light microscope and incubate the plate at 37 °C with 5% CO_2_.
  11. Perform medium change with 2 mL/well of 37 °C AMM (with growth factors) the next day followed by every second day.

## Validation of protocol

This protocol has been used and validated in the following research article:

- Stoklund Dittlau et al. [24]. *FUS*-ALS hiPSC-derived astrocytes impair human motor units through both gain-of-toxicity and loss-of-support mechanisms. Molecular Neurodegeneration (Figures 1, 2, 4–7, Supplemental figures 1–4 and 6–8, and Additional files 3–6).
  The protocol is an optimized version of our previous hiPSC-derived astrocyte protocol, which was used and validated in the following research article:
- Chandrasekaran et al. [25] Astrocyte reactivity triggered by defective autophagy and metabolic failure causes neurotoxicity in frontotemporal dementia type 3. Stem Cell Reports (Figures 1–5 and Supplemental figures 1–5).

## General notes and troubleshooting

**General notes**

1. Weekend work can be avoided by increasing medium volumes during medium changes on Fridays (4–6 mL/well for 6-well plates, 2–3 mL/well for 12-well plates, and 1 mL/well for 24-well plates). No addition of medium is required for flasks.
2. Always change the medium the day after passaging and thawing cells.
3. Increase passage number upon passaging, cryopreserving, and thawing.

**Troubleshooting**

Problem 1: EBs attach to the bottom of the T25 low-attachment flask around d4–d7.

Possible cause: EBs might be slightly too large or the T25 low-attachment flask might have a flaw in its surface treatment.

Solution: Transfer EBs in fresh NIM to a new T25 low-attachment flask. When transferring, pipette up and down 2–3 times to decrease the size of the EBs.

Problem 2: Complete cell death during d16-d25.

Possible cause: Cell density at d15 is too low.

Solution: Aim for >90% cell confluence at d15.

## References

[1] Masrori P. and Van Damme P. (2020). Amyotrophic lateral sclerosis: a clinical review. Eur. J. Neurol. 27(10): 1918 1929 1929. doi: 10.1111/ene.14393 32526057 PMC7540334

[2] Renton A. E., Chiò A. and Traynor B. J. (2014). State of play in amyotrophic lateral sclerosis genetics. Nat. Neurosci. 17(1): 17 23 23. doi: 10.1038/nn.3584 24369373 PMC4544832

[3] Tziortzouda P., L. Van Den Bosch and Hirth F. (2021). Triad of TDP43 control in neurodegeneration: autoregulation, localization and aggregation. Nat. Rev. Neurosci. 22(4): 197 208 208. doi: 10.1038/s41583-021-00431-1 33654312

[4] Fazal R., Boeynaems S., Swijsen A., De Decker M., Fumagalli L., Moisse M., Vanneste J., Guo W., Boon R., Vercruysse T., .(2021). HDAC6 inhibition restores TDP‐43 pathology and axonal transport defects in human motor neurons with *TARDBP* mutations. EMBO J. 40(7): e106177. doi: 10.15252/embj.2020106177 PMC8013789 33694180

[5] Guo W., Naujock M., Fumagalli L., Vandoorne T., Baatsen P., Boon R., Ordovás L., Patel A., Welters M., Vanwelden T., .(2017). HDAC6 inhibition reverses axonal transport defects in motor neurons derived from FUS-ALS patients. Nat. Commun. 8(1): 861. doi: 10.1038/s41467-017-00911-y 29021520 PMC5636840

[6] Fumagalli L., Young F. L., Boeynaems S., De Decker M., Mehta A. R., Swijsen A., Fazal R., Guo W., Moisse M., Beckers J., .(2021). *C9orf72*-derived arginine-containing dipeptide repeats associate with axonal transport machinery and impede microtubule-based motility. Sci. Adv. 7(15): eabg3013. doi: 10.1126/sciadv.abg3013 PMC8034861 33837088

[7] Naumann M., Pal A., Goswami A., Lojewski X., Japtok J., Vehlow A., Naujock M., Günther R., Jin M., Stanslowsky N., .(2018). Impaired DNA damage response signaling by FUS-NLS mutations leads to neurodegeneration and FUS aggregate formation. Nat. Commun. 9(1): 335. doi: 10.1038/s41467-017-02299-1 29362359 PMC5780468

[8] Qian K., Jiang X., Liu Z. Q., Zhang J., Fu P., Su Y., Brazhe N. A., Liu D. and Zhu L. Q. (2023). Revisiting the critical roles of reactive astrocytes in neurodegeneration. Mol. Psychiatry 28(7): 2697 2706 2706. doi: 10.1038/s41380-023-02061-8 37037874

[9] Verkhratsky A. and Nedergaard M. (2018). Physiology of Astroglia. Physiol. Rev. 98(1): 239 389 389. doi: 10.1152/physrev.00042.2016 29351512 PMC6050349

[10] Stoklund Dittlau K. and Van Den Bosch L. (2023). Why should we care about astrocytes in a motor neuron disease?. Front. Mol. Med. 3: 1047540. doi: 10.3389/fmmed.2023.1047540 PMC11285655 39086676

[11] Petrov D., Mansfield C., Moussy A. and Hermine O. (2017). ALS Clinical Trials Review: 20 Years of Failure. Are We Any Closer to Registering a New Treatment? Front. Aging Neurosci. 9: 68. doi: 10.3389/fnagi.2017.00068 28382000 PMC5360725

[12] Oberheim N. A., Takano T., Han X., He W., Lin J. H. C., Wang F., Xu Q., Wyatt J. D., Pilcher W., Ojemann J. G., .(2009). Uniquely Hominid Features of Adult Human Astrocytes. J. Neurosci. 29(10): 3276 3287 3287. doi: 10.1523/jneurosci.4707-08.2009 19279265 PMC2819812

[13] Allen N. J. and Eroglu C. (2017). Cell Biology of Astrocyte-Synapse Interactions. Neuron 96(3): 697 708 708. doi: 10.1016/j.neuron.2017.09.056 29096081 PMC5687890

[14] Bushong E. A., Martone M. E., Jones Y. Z. and Ellisman M. H. (2002). Protoplasmic Astrocytes in CA1 Stratum Radiatum Occupy Separate Anatomical Domains. J. Neurosci. 22(1): 183 192 192. doi: 10.1523/jneurosci.22-01-00183.2002 11756501 PMC6757596

[15] Birger A., Ben-Dor I., Ottolenghi M., Turetsky T., Gil Y., Sweetat S., Perez L., Belzer V., Casden N., Steiner D., .(2019). Human iPSC-derived astrocytes from ALS patients with mutated C9ORF72 show increased oxidative stress and neurotoxicity. eBioMedicine 50: 274 289 289. doi: 10.1016/j.ebiom.2019.11.026 31787569 PMC6921360

[16] Hedegaard A., Monzón-Sandoval J., Newey S. E., Whiteley E. S., Webber C. and Akerman C. J. (2020). Pro-maturational Effects of Human iPSC-Derived Cortical Astrocytes upon iPSC-Derived Cortical Neurons. Stem Cell Rep. 15(1): 38 51 51. doi: 10.1016/j.stemcr.2020.05.003 PMC7363746 32502466

[17] Mulica P., Venegas C., Landoulsi Z., Badanjak K., Delcambre S., Tziortziou M., Hezzaz S., Ghelfi J., Smajic S., Schwamborn J., .(2023). Comparison of two protocols for the generation of iPSC-derived human astrocytes. Biol. Proced. Online 25(1): 26. doi: 10.1186/s12575-023-00218-x 37730545 PMC10512486

[18] Krencik R. and Zhang S. C. (2011). Directed differentiation of functional astroglial subtypes from human pluripotent stem cells. Nat. Protoc. 6(11): 1710 1717 1717. doi: 10.1038/nprot.2011.405 22011653 PMC3198813

[19] Perriot S., Mathias A., Perriard G., Canales M., Jonkmans N., Merienne N., Meunier C., El Kassar L., Perrier A. L., Laplaud D. A., .(2018). Human Induced Pluripotent Stem Cell-Derived Astrocytes Are Differentially Activated by Multiple Sclerosis-Associated Cytokines. Stem Cell Rep. 11(5): 1199 1210 1210. doi: 10.1016/j.stemcr.2018.09.015 PMC6234919 30409508

[20] Perriot S., Canales M., Mathias A. and Du Pasquier R. (2021). Differentiation of functional astrocytes from human-induced pluripotent stem cells in chemically defined media. STAR Protoc. 2(4): 100902. doi: 10.1016/j.xpro.2021.100902 34746863 PMC8551928

[21] Peteri U. K., Pitkonen J., Utami K. H., Paavola J., Roybon L., Pouladi M. A. and Castrén M. L. (2021). Generation of the Human Pluripotent Stem-Cell-Derived Astrocyte Model with Forebrain Identity. Brain Sciences 11(2): 209. doi: 10.3390/brainsci11020209 33572154 PMC7914711

[22] Chandrasekaran A., Avci H. X., Ochalek A., Rösingh L. N., Molnár K., László L., Bellák T., Téglási A., Pesti K., Mike A., .(2017). Comparison of 2D and 3D neural induction methods for the generation of neural progenitor cells from human induced pluripotent stem cells. Stem Cell Res. 25: 139 151 151. doi: 10.1016/j.scr.2017.10.010 29128818

[23] Shaltouki A., Peng J., Liu Q., Rao M. S. and Zeng X. (2013). Efficient Generation of Astrocytes from Human Pluripotent Stem Cells in Defined Conditions. Stem Cells 31(5): 941 952 952. doi: 10.1002/stem.1334 23341249

[24] Stoklund Dittlau K., Terrie L., Baatsen P., Kerstens A., De Swert L., Janky R., Corthout N., Masrori P., Van Damme P., Hyttel P., .(2023). FUS-ALS hiPSC-derived astrocytes impair human motor units through both gain-of-toxicity and loss-of-support mechanisms. Mol. Neurodegener. 18(1): 5. doi: 10.1186/s13024-022-00591-3 36653804 PMC9847053

[25] Chandrasekaran A., Stoklund Dittlau K., Corsi G. I., Haukedal H., Doncheva N. T., Ramakrishna S., Ambardar S., Salcedo C., Schmidt S. I., Zhang Y., .(2021). Astrocytic reactivity triggered by defective autophagy and metabolic failure causes neurotoxicity in frontotemporal dementia type 3. Stem Cell Rep. 16(11): 2736 2751 2751. doi: 10.1016/j.stemcr.2021.09.013 PMC8581052 34678206

